# High Flow, High-Pressure Retrograde Cerebral Perfusion at 28°C is Safe and Effective for Hemiarch Replacement of the Ascending Aorta

**DOI:** 10.1055/a-2564-0323

**Published:** 2025-05-02

**Authors:** R. Wilson King, Adam M. Carroll, Michal Schäfer, Zihan Feng, Jintong W. Liu, George A. Justison, Joseph C. Cleveland, Jessica Y. Rove, Muhammad Aftab, T. Brett Reece

**Affiliations:** 1 Department of Surgery, University of Colorado, Denver, Colorado; 2 Department of Cardiothoracic Surgery, University of Colorado, Denver, Colorado

**Keywords:** retrograde cerebral perfusion, antegrade cerebral perfusion, circulatory arrest, elective hemiarch

## Abstract

**Background/Objective:**

Traditional retrograde cerebral perfusion (RCP) parameters may be suboptimal for washout of debris during hemiarch replacement of the ascending aorta, so we have designed a protocol of increased RCP pressure and flow at moderate hypothermia. We hypothesize that higher RCP pressure is safe in neurological outcomes in cases utilizing circulatory arrest at 28°C in elective hemiarch replacement.

**Methods:**

A retrospective review of a single-institution prospective database was used to search for all patients with elective hemiarch surgery from 2015 to 2022. Two cohorts were created—patients who received RCP only during circulatory arrest at 28°C and patients who received selective antegrade cerebral perfusion (SACP) during circulatory arrest. Neurological and postoperative outcomes were compared. Arterial blood gas measurements during RCP were taken from the left carotid of 34 patients, which were compared with the arterial blood gas from the bypass circuit to ensure adequate oxygen extraction. Propensity score matching was used to adjust for perioperative indices and patient characteristics.

**Results:**

A total of 248 patients were in the SACP cohort and 79 patients in the RCP cohort. The two groups were similar based on patient demographics and relevant comorbidities. The cohorts differed in nadir bladder temperature, circulatory arrest time, and cardiopulmonary bypass time. After propensity matching, nadir bladder temperature, circulatory arrest, and cardiopulmonary bypass times were similar. Neurological postoperative outcomes were similar in the unmatched and matched analysis. The median pressure in the RCP group during circulatory arrest was 40 mm Hg. The median change in oxygen from bypass circuit to the carotids is 398 mm Hg with a mean oxygen extraction of 93.3%.

**Conclusion:**

These data demonstrate that a more aggressive approach to RCP beyond traditional constraints at 28°C is safe for short periods of circulatory arrest. Even with the new RCP parameters and after adjusting for standard patient and perioperative characteristics, there is no difference between SACP and RCP in neurological outcomes. Further, adequate oxygen extraction is achieved during RCP.

## Introduction

The prevalence of ascending aortic aneurysms and dissections has increased, leading to an increase in the number of surgical interventions. 1 Many different cerebral perfusion strategies, including retrograde cerebral perfusion (RCP) and selective antegrade cerebral perfusion (SACP), during the requisite circulatory arrest period have been utilized, with much debate regarding the most effective modality, 2 although a few recent studies have shown no difference between the two modalities. 3 4 5

During the earlier years of RCP use, many thought that standard RCP parameters could lead to cerebral edema and thus poor postoperative neurological outcomes 6 when compared with SACP. Furthermore, there are concerns about the nonphysiological blood flow, due to the retrograde manner of flow, causing poor oxygen extraction, and poor flow through the capillaries. 7 8 However, many surgeons prefer to use RCP as an adjunct to deep or moderate hypothermic circulatory arrest (HCA) because of the potential to washout embolic debris, thus decreasing the risk of embolic stroke.

Our institution has adopted an RCP only protocol that utilizes higher flow and higher pressure RCP than standard parameters at moderate hypothermia during circulatory arrest. This study aims to compare this protocol in patients undergoing elective hemiarch replacement of the aorta to patients who receive antegrade cerebral perfusion (ACP) only in terms of postoperative neurological outcomes.

## Materials and Methods

### Study Design

A retrospective review of a single-institution prospective database with consecutive patients who received aortic surgery was used to search for all patients who received elective hemiarch replacement of the aorta from 2015 to 2022. The indication for hemiarch replacement was for ascending aortic aneurysmal disease. Using these patients, two cohorts were created—patients who received RCP only at 28°C during the requisite circulatory arrest time and patients who received SACP only during the requisite circulatory arrest time. This study does not include any patients that received both RCP and SACP for circulatory arrest, and it does not include any patients who had operative indications other than ascending aneurysmal disease. Intraoperatively, the RCP only cohort had increasing flows and pressure (jugular venous pressure measured from the central line) up to 50 mm Hg or until blood was found to be returned from the carotids. Further, arterial blood gas (ABG) measurements were taken during the run of RCP from the left carotid and the bypass circuit and the ratio used to roughly calculate the oxygen extraction to ensure adequate oxygen extraction in 34 of these patients. RCP pressures were also recorded from the RCP circuit to quantify these higher pressure parameters. This study was approved by the University of Colorado Institutional Review Board with waived written consent as this is an institutionally approved database of all patients undergoing cardiac surgery.

### Circulatory Arrest Cannulation Techniques

The two cannulation strategies analyzed in this study are SACP and RCP. For SACP, the innominate artery is cannulated directly with a 10-mm graft anastomosis or by access with a 14-Fr arterial cannula through a purse string using the modified Seldinger technique. The flows are started at 10 to 15 mL/kg and increased if cerebral oximetry drops on the contralateral side. For RCP, a Rummel tourniquet is placed above the azygos vein in the superior vena cava (SVC). A 24-Fr cannula is purse-stringed directly into the SVC. The flows are started at 10 mL/kg until backflow is visualized from the carotids. This is dictated by surgeon visualization of blood return from the carotid.

### Statistical Analysis

Statistical analyses and data presentation were performed using SAS and Prism (version 9.0 or higher, GraphPad Software Inc., La Jolla, CA). All considered variables were checked for the distributional assumption of normality using normal plots, in addition to D'Agostino-Pearson, Shapiro–Wilk, and Kolmogorov–Smirnov tests. Demographic and clinical characteristics are summarized with descriptive statistics (mean and standard deviation, median and 25th/75th percentiles, frequencies, and percentages).

Demographic and clinical characteristics among RCP and SACP patients were compared using the unpaired *t* -tests, Mann–Whitney test, chi-square, or Fisher's exact tests as dictated by the nature of sampled variables. The primary outcome measures were postoperative stroke and delirium. Postoperative stroke was defined as an imaging-confirmed stroke with persistent neurological deficits. Delirium was defined as mental status disturbances including hypoactive and hyperactive states with confusion that is reversible. This endpoint was included if documented by board-certified critical care physicians, residents, or advanced practice providers. Secondary outcome measures include standard postoperative indices for cardiothoracic surgery. A 1:1 propensity score matching was used to adjust for perioperative and patient characteristics. The probability of undergoing SACP versus RCP was calculated by a multivariable logistic regression model that contained all perioperative (cross-clamp time, bypass time, and circulation arrest time) and patient characteristics (age, sex, and history of prior surgery). A caliper of 0.01 on the propensity score scale was used for nearest neighbor matching with a 1:1 ratio without replacement. Demographic and clinical characteristics among SACP and RCP were compared using the unpaired *t* -tests, Mann–Whitney test, chi-square, or Fisher's exact tests as dictated by the nature of sampled variables. Simple linear regression using Pearson *R* was applied to test the association between the aortic size and stiffness indices. *p* -Values were determined to be significant at an α level < 0.05.

## Results

A total of 327 patients underwent elective hemiarch replacement of the ascending aorta during the years of 2015 to 2022, who received either RCP only or SACP only during circulatory arrest. There were 248 patients (75.8%) who received SACP only and 79 patients (24.2%) who received RCP only. See Table 1 for patient characteristics in both groups before propensity matching. Of note, there were no significant differences in the SACP and RCP groups in terms of patient demographics and preoperative characteristics except for more smoking in the SACP group. Intraoperatively, however, the RCP group had a lower median cardiopulmonary bypass time (125 vs. 138 minutes; *p* = 0.03) and a lower median circulatory arrest time (6 vs. 10 minutes, *p* < 0.001). Furthermore, the RCP only group had more additional aortic valve procedures and a higher nadir bladder temperature (28.0 vs. 27.6°C, *p* = 0.02). See Table 2 for intraoperative characteristics. Before propensity matching, there were no significant differences in the incidence of postoperative stroke or postoperative delirium; however, there was an increased intensive care unit (ICU) length of stay (LOS) in the RCP only group ( Table 3 ). Postoperative acute hypoxic respiratory failure, bleeding, and acute kidney injury were similar in both groups and at low rates (all less than 2%).

**Table 1:** Preoperative patient characteristics

	SACP only	RCP only	*p* -Value
*N*	248	79	
Age	62 (50–70)	60 (47–70)	0.80
Sex (female)	61 (24.6%)	19 (24.0%)	0.99
Race			
Caucasian	204 (82.3%)	61 (77.2%)	0.99
African American	13 (5.2%)	5 (6.3%)	0.71
Hispanic	22 (8.9%)	10 (12.7%)	0.97
Asian	6 (2.4%)	1 (1.3%)	0.54
Other	3 (1.2%)	2 (2.5%)	0.83
Comorbidities			
Dyslipidemia	102 (41.1%)	27 (34.1%)	0.27
Hypertension	162 (65.3%)	45 (57.0%)	0.09
Smoking	78 (31.5%)	10 (12.7%)	0.01 a
Diabetes	30 (12.1%)	8 (10.1%)	0.89
Renal disease	19 (7.7%)	9 (11.4%)	0.30
Obesity	40 (16.1%)	16 (20.3%)	0.40
Stroke	14 (5.6%)	4 (5.1%)	0.84
BMI (kg/m ^2^ )	27.7 (24.9–31.9)	26.8 (23.9–31.2)	0.55
Redo sternotomy	63 (25.4%)	20 (30.4%)	0.99

Abbreviations: BMI, body mass index; RCP, retrograde cerebral perfusion; SACP, selective antegrade cerebral perfusion.

a Statistical significance.

**Table 2:** Intraoperative characteristics

	SACP only	RCP only	*p* -Value
*N*	248	79	
Additional interventions			
Aortic valve repair/replacement	89 (36.3%)	35 (44.3%)	0.025 a
Aortic root replacement	105 (42.5%)	26 (32.9%)	0.14
Cardiopulmonary bypass time (min)	138 (115–175)	125 (109–159)	0.03 a
Cross-clamp time (min)	95 (71–125)	97 (79–124)	0.54
Circulatory arrest time (min)	10 (7–12)	6 (5–8)	<0.001 a
Nadir bladder temperature (°C)	27.6 (26.3–28.0)	28.0 (26.7–28.1)	0.02 a
Intraoperative blood products			
Red blood cells	0 (0–0)	0 (0–0)	
Fresh-frozen plasma	2 (0–4)	0 (0–2)	
Cryoprecipitate	0 (0–0)	0 (0–0)	
Platelets	1 (0–2)	0 (0–1)	

Abbreviations: RCP, retrograde cerebral perfusion; SACP, selective antegrade cerebral perfusion.

a Statistical significance.

**Table 3:** Postoperative outcomes

	SACP only	RCP only	*p* -Value
*N*	248	79	
ICU length of stay (d)	3 (2–4)	2 (1–3)	0.03 a
Stroke	6 (2.4%)	4 (5%)	0.26
Delirium	17 (6.8%)	4 (5.0%)	0.79

Abbreviations: ICU, intensive care unit; RCP, retrograde cerebral perfusion; SACP, selective antegrade cerebral perfusion.

a Statistical significance.

Because the groups differed in intraoperative characteristics, propensity matching was used to create two cohorts of 60 patients in both the RCP only and SACP only groups. After propensity matching, both groups had similar preoperative patient characteristics and intraoperative characteristics. When adjusted for standard patient and perioperative characteristics, there is no difference between the SACP only RCP only cohorts in terms of neurological outcomes. Importantly, the postoperative stroke incidence in the RCP only and SACP group were similar (3.3 vs. 3.3%, *p* = 0.99), and the postoperative ICU delirium in both RCP and SACP groups were similar (6.6 vs. 3.3%, *p* = 0.68). See Table 4 for the analysis before and after propensity matching. Of note, there was zero 30-day mortality in both matched groups.

**Table 4:** Before and after propensity matching: intraoperative characteristics and postoperative outcomes

	Before matching			After matching		
	SACP ( *n* = 248)	RCP ( *n* = 79)	*p* -Value	SACP ( *n* = 60)	RCP ( *n* = 60)	*p* -Value
Age (y)	62 (50–70)	60 (47–70)	0.80	62 (49–9)	59 (46–69)	0.55
Sex (female)	61 (24.6%)	19 (24.0%)	0.99	16 (26.7%)	17 (28.3%)	0.99
Redo sternotomy	63 (25.4%)	20 (30.4%)	0.99	23 (21.7%)	19 (32.7%)	0.30
BMI (kg/m ^2^ )	27.7 (25–32)	26.8 (24–31)	0.55	62 (48–69)	59 (46–69)	0.42
CPB (min)	138 (115–175)	125 (109–159)	0.03	121 (102–145)	124 (109–170)	0.35
XC (min)	95 (71–125)	97 (79–124)	0.54	90 (64–111)	97 (79–115)	0.12
Nadir bladder temperature	27.6 (26.3–28.0)	28.0 (26.7–28.1)	0.02	27.5 (26.5–28.0)	28.0 (27.1–28.2)	0.07
Circ arrest time (min)	10 (7–12)	6 (5–8)	<0.001	7 (5–9)	6 (5–8)	0.34
Hospital LOS (days)	7 (6–9)	7 (6–8)	0.84	7 (6–9)	7 (6–8)	0.90
ICU LOS (d)	3 (2–4)	2 (1–3)	0.03	2 (1–3)	2 (1–3)	0.61
Stroke	6 (2.4%)	4 (5.0%)	0.26	2 (3.3%)	2 (3.3%)	0.99
Delirium	17 (6.8%)	4 (5.0%)	0.79	2 (3.3%)	4 (6.6%)	0.68

Abbreviations: BMI, body mass index; CPB, cardiopulmonary bypass; Circ, circulatory; ICU, intensive care unit; LOS, length of stay; RCP, retrograde cerebral perfusion; SACP, selective antegrade cerebral perfusion; XC, cross-clamp.

### Retrograde Cerebral Perfusion Parameters

During circulatory arrest, RCP flows and pressure were increased until visualization of return of blood flow from the carotids. Thirty-four of our sampled RCP only patients had ABG taken from the left carotid and the bypass circuit to ensure adequate oxygen extraction. Furthermore, RCP pressure measurements were also made to confirm the higher pressure used during circulatory arrest. The median change in PaO _2_ was 398 mm Hg (interquartile range [IQR]: 297.5, 452 mm Hg) for a median oxygen extraction of 93.3% (IQR: 91.8, 95.3%). Maximum RCP pressures were also determined. The median of the maximum RCP pressures was 40 mm Hg (IQR: 31, 52).

## Discussion

**Fig. 1:**
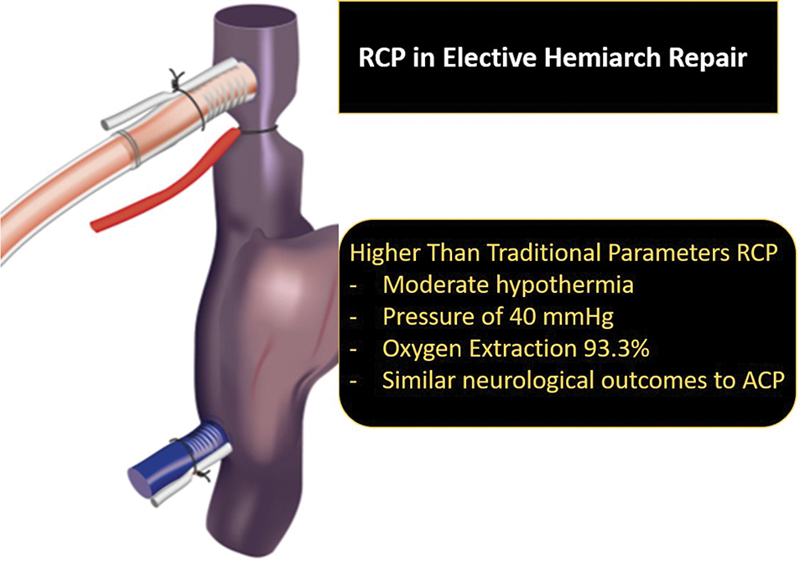
Retrograde cerebral perfusion at higher than traditional parameters leads to similar neurological outcomes to antegrade cerebral perfusion in elective hemiarch repair.

These data suggest that using RCP at a higher flow and higher pressure (median 40 mm Hg [IQR: 31–52]) is safe in terms of neurological outcomes ( Fig. 1 ). The incidence of postoperative stroke (3.3%) and postoperative delirium (6.6%) was comparable to the SACP only cohort before and after propensity matching. Furthermore, there were similar postoperative outcomes in terms of mortality and LOS.

Much debate surrounds the best optimal cerebral protection strategy during circulatory arrest in aortic surgery. At our institution, if the circulatory arrest time is less than 10 minutes, almost all of our patients receive RCP only because of the theoretical benefit of embolic debris washout. Because of this, our RCP only cohort had shorter circulatory arrest times than the SACP only cohort. This made propensity matching necessary for optimal comparison between the SACP only cohort and the RCP only cohort. After matching, postoperative outcomes remained similar suggesting these higher parameters are safe when undergoing elective hemiarch repair of the aorta.

For our institutions' total arch replacements, we use a head-first approach, which allows us to aim for a less than 20-minute circulatory arrest time using RCP. If there is a more complex reconstruction needed, we then use bilateral ACP through the head graft for the remaining period. However, the goal remains to use RCP as the initial strategy.

RCP was first suggested to treat air embolism and has since evolved as a standard adjunct to HCA for cerebral protection for the benefit of stroke prevention. 9 In animal models, there have been suggestions that RCP delivers blood flow and oxygen at a lower rate than ACP flow, but many of these models were published more than 20 years ago. 7 10 11 However, these concerns do not seem to translate clinically, as many studies have shown the benefit, or at least the neutrality, of RCP. 12 13 14 Our study indicates that the RCP flow, at pressures of 40 mm Hg, does lead to more than adequate oxygen extraction by cerebral tissue (median: 93% oxygen extraction). Furthermore, our group has previously published an analysis of patients receiving RCP + ACP compared with ACP only, which showed similar results regarding postoperative neurological outcomes. 15 That study led to the current study comparing RCP only without ACP to ACP, this study has more specific details regarding oxygen extraction and actual pressures of the RCP circuit. Furthermore, this study uses propensity-matched analysis to compare similar groups.

Much debate also exists regarding the levels of cerebral edema that occur due to higher flow pressures in the RCP circuit. Animal studies suggest that pressures above 30 mm Hg lead to significant cerebral edema. 16 It seems that many surgeons prefer RCP pressure not to exceed 25mm Hg because of these concerns. 17 In fact, a prior propensity-matched analysis has shown similar stroke and mortality rates with RCP and ACP, but increased transient neurological deficit in the RCP group, further highlighting these concerns. 18 However, our data indicate that we have similar rates of postoperative delirium and stroke suggesting that the cerebral edema concerns may be overwrought and deserve further analysis.

The final point to discuss is that our mortality and stroke rates are similar in the RCP only group and the ACP only group. Both matched groups had similar stroke rates and zero 30-day mortality. Although the hope is to see a reduction in stroke rates and thus improved mortality rates, these data did not support that finding. It is important to note that prior studies trying to prove or disprove this theory have been mixed. For example, a meta-analysis in 2014 found that there was no statistical significant difference in ACP and RCP in terms of 30-day mortality, stroke, and transient neurological dysfunction. 5 In 2016, a study examined RCP versus ACP and found a benefit for the ACP group in terms of neurological complications, as well as a trend toward decreased mortality. 19 A more recent small prospective analysis in 2019 found that RCP patients with deep HCA compared with ACP with moderate HCA were similar in terms of mortality but the ACP + moderate HCA group had a higher rate of brain lesions on imaging. 12 Importantly, however, our study had patients who were under moderate hypothermia and higher RCP pressures and showed similar mortality and neurological dysfunction rates. This highlights the need for randomized trials to help determine the optimal cerebral protection strategies in different patient populations and operations.

### Limitations

The limitation to this study was that it is a retrospective review of a single high-volume aortic center with much experience in RCP, use which may impact the generalizability of this study. Being a high-volume aortic center, we acknowledge that our circulatory arrest times are short, which could explain some of our beneficial findings and make them less generalizable to centers with longer circulatory arrest times. Further, most of the RCP only patients were from more recent years, based on the evolution of practice at our institution, which may impact the study. Finally, our study does not make any conclusions regarding all neurological deficits, as we do not routinely employ imaging that could detect subclinical infarcts.

## Conclusion

Open aortic surgery, including elective hemiarch replacement of the ascending aorta, carries the risks of stroke and mortality. The debate is open on the optimal cerebral perfusion strategies during circulatory arrest. Many institutions that use RCP as an adjunct in circulatory arrest use deep hypothermia and more standard RCP parameters. This propensity-matched analysis in patients undergoing hemiarch replacement of the aorta at moderate hypothermia and higher than standard RCP parameters suggests that RCP at moderate hypothermia is safe and effective in terms of mortality and neurological dysfunction. Although these findings are preliminary in nature, these data highlight the need for randomized trials to help determine the best strategy for these challenging patients.
